# Analyzing body composition in living kidney donors: impact on post-transplant kidney function

**DOI:** 10.3389/fneph.2024.1467669

**Published:** 2024-11-25

**Authors:** Evelien E. Quint, Lisa B. Westenberg, Gertrude J. Nieuwenhuijs-Moeke, Eva A. N. van den Broek, Marcel Zorgdrager, Alain R. Viddeleer, Stephan J. L. Bakker, Ija M. Nolte, Marco van Londen, Robert A. Pol, TransplantLines Investigators

**Affiliations:** ^1^ Department of Surgery, Division of Transplantation Surgery, University Medical Center Groningen, University of Groningen, Groningen, Netherlands; ^2^ Department of Anesthesiology, University of Groningen, University Medical Centre Groningen, Groningen, Netherlands; ^3^ Department of Radiology, Medical Imaging Center, University Medical Center Groningen, University of Groningen, Groningen, Netherlands; ^4^ Department of Internal Medicine, Division of Nephrology, University Medical Center Groningen, University of Groningen, Groningen, Netherlands; ^5^ Department of Epidemiology, University Medical Center Groningen, University of Groningen, Groningen, Netherlands

**Keywords:** living donor kidney transplantation, body composition, kidney function, body mass index, computed tomography

## Abstract

Living donor kidney transplantation boasts superior patient and graft survival rates compared to deceased donor kidney transplantation. However, the impact of living donor body composition (BC) on post-transplant kidney function remains uncertain. In a cohort of 293 living kidney donor-recipients pairs, we utilized linear mixed model analyses, adjusted for time and including a multiplicative interaction term of time with the donor body composition measure, and found no significant associations between any donor BC measure and the annual change in recipient post-transplantation estimated glomerular filtration rate (eGFR) [donor body mass index (BMI): *B*=-0.01, 95%CI -0.13; 0.11, *p*=0.88; donor waist circumference: *B*=0.02, 95%CI -0.02; 0.06, *p*=0.38; donor skeletal muscle index: *B*=-0.02, 95%CI -0.07; 0.04, *p*=0.63; donor skeletal muscle radiation attenuation: *B*=-0.002, 95%CI -0.06; 0.06, *p*=0.96; donor visceral adipose tissue index: *B*=-0.001, 95%CI -0.02; 0.02, *p*=0.93; donor subcutaneous adipose tissue index: *B*=-0.001, 95%CI -0.02; 0.02, *p*=0.94; donor intramuscular adipose tissue index: *B*=-0.12, 95%CI -0.29; 0.06, *p*=0.19; donor total abdominal adipose tissue index: *B*=-0.001, 95%CI -0.01; 0.01, *p*=0.89]. Our study suggests that pre-donation BC does not affect post-transplantation recipient eGFR in donor populations with a BMI below 35 kg/m^2^.

## Introduction

Kidney transplantation (KT) remains the preferred treatment for patients with end stage kidney disease (ESKD) (1). Although living donor kidney transplantation (LDKT) is less common than deceased donor kidney transplantation (DDKT), it has important advantages in terms of patient and graft survival compared to DDKT (2–4). To ensure the safety and efficacy of LDKT, potential donors undergo a thorough screening process to assess potential risk factors for adverse outcomes of donation. Among these factors, obesity emerges as an important concern due to its association with decreased long-term kidney function through conditions such as hypertension, diabetes, and metabolic syndrome, which can ultimately lead to the development of Chronic Kidney Disease (CKD) and/or ESKD (5, 6). Importantly, donor obesity potentially also impacts recipients (7). KT recipients who receive a kidney from an obese living donor may experience a slight increase in the risk of perioperative complications, delayed graft function and kidney graft loss (6, 8–10). Due to the donor obesity-related health risks for both the donor and the recipient, many centers impose a body mass index (BMI) above 30 kg/m^2^ as a relative contra-indication and above 35 kg/m^2^ as a hard contra-indication for living kidney donation (11). However, recognizing the inherent limitations of BMI, including its inability to distinguish between muscle mass and fat mass and to differentiate subcutaneous fat from visceral fat, the need of exploring alternative body composition metrics arises. New techniques including radiological imaging have proven to be more precise in measuring different fat and muscle compartments (12). Several KT recipient body composition measures have been linked to adverse post-KT outcomes, resulting in reduced graft function, reduced graft survival and an increased risk of post-KT mortality (13–15). However, little is known about the effect of living kidney donor body composition on post-KT outcomes. An excess of donor fat tissue may have led to direct injury of the donor kidney prior to donation through processes involving increased numbers of adipose-derived molecules and dysregulated metabolites, leading to oxidative stress, inflammation, and kidney fibrosis (16). It may also be possible that, in an effort to meet the increased metabolic demands of the body, hyperfiltration may have occurred prior to donation in overweight or obese donors (17). Glomerular hyperfiltration has been associated with kidney function decline and the development of CKD (18). Transplantation of such a kidney from a donor with a suboptimal body composition type may affect post-transplantation kidney function in the recipient.

Hence, the goal of this study was to determine the association between living donor body composition, evaluated through conventional measures and computed tomography (CT), and the change in recipient estimated glomerular filtration rate (eGFR) after transplantation. We hypothesized that higher donor BMI, higher waist circumference, and higher values of radiologic adipose tissue measurements are associated with a greater negative change in recipient eGFR after transplantation. Furthermore, we hypothesized that age has an impact on recipient eGFR after transplantation.

## Method

### Study design and population

In total, 293 pairs of living kidney donors and kidney transplant recipients were included in this study. All transplantations took place between 2002 and 2019 at the University Medical Center Groningen (UMCG, Groningen, the Netherlands). The data were sourced from the TransplantLines Biobank and Cohort Study (ClinicalTrials.gov identifier: NCT03272841), an ongoing, prospective study aiming to assess short- and long-term outcomes in solid organ transplant donors and recipients (19). As part of this study, kidney transplant recipients had their estimated glomerular filtration rate (eGFR) measured at predefined timepoints following transplantation: 3 months, 6 months, 1 year, 2 years, 5 years, 10 years, and then at subsequent five-year intervals.

All solid organ transplantation donors and recipients (aged ≥18 years) were invited to participate and gave written informed consent on enrolment. The study protocol was approved by the local institutional ethical review board (METc 2014/077). All procedures were conducted in accordance with the declaration of Helsinki and declaration of Istanbul. Study-specific exclusion criteria (in addition to those described in the TransplantLines protocol) were the presence of significant interfering artefacts on CT imaging and/or incomplete visualization of the abdominal wall muscles and/or subcutaneous fat tissue on the CT scan.

### Data collection

All donors underwent CT imaging as an integral part of the screening protocol for living kidney donation at the UMCG, with the majority (n=292) conducted at the UMCG and one at a non-academic referring hospital in the Netherlands. CT scans were predominantly contrast-enhanced (n=292) (n=2 portal venous phase, n=4 arterial phase, n=286 late phase) and the remaining one was unenhanced. Most scans had a slice thickness of 2 mm (n=284, 97%). Tube voltage ranged from 80-140 kVp (median 100 [IQR 90-100]) and the current varied from 20-455 mAs (mean 89.7 ± 48.1 mAs). For analysis scans at the level of the third lumbar vertebra (L3) were used, which have been shown to correspond with total body mass of skeletal muscle, subcutaneous adipose tissue (SAT) and visceral adipose tissue (VAT) (20). Voxels with densities ranging from -29 to +150 Hounsfield units (HU) indicated muscle tissue and were selected to measure the psoas, paraspinal, and abdominal wall muscles. The skeletal muscle area (SMA, cm^2^) was determined and indexed for donor height^2^ (skeletal muscle index, SMI, cm^2^/m^2^) in accordance with a previous publication by our group (21). Fat tissue, defined by voxels ranging from -190 to -30 HU was assessed for SAT, VAT, and intramuscular adipose tissue (IMAT). Total abdominal adipose tissue (TAT) was calculated as SAT + VAT + IMAT. All adipose tissue measurements were indexed for donor height^2^ (m^2^) and yielded the subcutaneous adipose tissue index (SATi), visceral adipose tissue index (VATi), intramuscular adipose tissue index (IMATi), and total abdominal adipose tissue index (TATi). All clinical and biochemical parameters were conducted following the protocols outlined in a previous publication (19). eGFR was calculated according to the Chronic Kidney Disease Epidemiology Collaboration (CKD-EPI) equation from 2021 (22).

### Statistical analyses

Categorical variables are presented as numbers and percentages and normally distributed continuous variables are presented as a mean ± standard deviation. Linear mixed model analysis was used to investigate possible associations between donor body composition measurements, and recipient post-transplant kidney function over time. This included subgroups of donors with a normal/overweight BMI and high/low intramuscular adipose tissue. Donor body composition measurements were considered fixed effects, as well as their interaction with time (defined as donor body composition measurement * time). The analyses were adjusted for donor age, donor sex, donor pre-donation measured GFR (mGFR) recipient age, and recipient sex, as well as multiplicative interaction terms of these variables with time. The “unstructured” covariance structure was used. The use of “compound symmetry” and “autoregressive” covariance structures did not change the results. Sensitivity analyses were performed in a subset of patients of whom multiple post-transplant eGFR assessments were available. Two-tailed values of p < 0.05 were considered to indicate statistical significance. Statistical analyses were performed using RStudio (PBC, Boston, MA, USA, 2021) and SPSS version 28.0 (IBM, Armonk, USA).

## Results

### Characteristics of the study population

A total of 293 donor-recipient pairs were included in this study (**Table 1**). Donor age was 53 ± 11 years and 41% of donors was female. Donor weight was 79.7 ± 12.6 kg, height was 177 ± 10 cm, and BMI was 25.2 ± 3.0 kg/m^2^. SMI was 48.3 ± 8.1 cm^2^/m^2^, TATi was 96.8 ± 39.7 cm^2^/m^2^, and mGFR at screening for donation was 113.0 ± 22.2 mL/min. Seventy eight donors were classified as being overweight and 70 donors were classified as being within a normal range (**Supplementary Table 6**). Recipient age at transplantation was 50 ± 13 years, and 42% of recipients were female. Recipient weight was 81.4 ± 15.6 kg, height was 174 ± 10 cm, and BMI was 26.9 ± 4.6 kg/m^2^. The majority of recipients had primary glomerular kidney disease (21.8%), followed by polycystic kidney disease (21.5%), renovascular disease (7.5%), and glomerulonephritis (6.8%). Two hundred and fifty-nine (88.4%) recipients had hypertension prior to transplantation and 41 (14.0%) had pre-existing diabetes mellitus manifested before transplantation. Twenty (6.8%) and 15 (5.2%) experienced delayed graft function and acute rejection, respectively. **Supplementary Figure S1** depicts the amount of consecutive post-transplant eGFR measurements per recipient. Median time between transplantation and post-transplant eGFR measurement was 1 year [interquartile range: 0-14 years].

**Table 1:** Baseline characteristics of the study population.

Variable	Number (%), mean ± SD or median [IQR]
Number of participants	
Donors	293
Recipients	293
Donor characteristics	
Age (years)	53 ± 11
Women (n, %)	119 (40.6%)
Body composition	
BMI (kg/m^2^)	25.2 ± 3.0
Waist circumference (cm)	89.8 ± 9.8
SMI (cm^2^/m^2^)	48.3 ± 8.1
SMRA (Hounsfield Units)	50.4 ± 6.8
VATi (cm^2^/m^2^)	39.9 ± 24.1
SATi (cm^2^/m^2^)	52.6 ± 25.1
IMATi (cm^2^/m^2^)	4.4 ± 2.2
TATi (cm^2^/m^2^)	96.8 ± 39.7
mGFR (ml/min)	113.0 ± 22.2
Recipient characteristics	
Age at transplantation (years)	50 ± 13
Women	124 (42.3%)
eGFR follow-up (years)	3.88 ± 1.86
Hypertension	259 (88.4%)
Diabetes Mellitus	41 (14.0%)
BMI (kg/m^2^)	26.9 ± 4.6
Etiology of kidney disease	
Primary glomerular disease	64 (21.8%)
Glomerulonephritis	20 (6.8%)
Polycystic kidney disease	63 (21.5%)
Renovascular	22 (7.5%)
Diabetes	15 (5.1%)
Dysplasia	14 (4.8%)
Other/unknown	95 (32.4%)
Cold ischemia time (minutes)*	157 [37.50]
1^st^ warm ischemia time (minutes)	3 [1]
2^nd^ warm ischemia time (minutes)*	40 [12]
Delayed graft function	20 (6.8%)
Acute rejection	15 (5.2%)

*in case of more than one cold ischemia time and/or more than two warm ischemia times, additional minutes were added to the cold ischemia time and 2^nd^ warm ischemia time.

### Donor body composition measurements and recipient post-transplantation eGFR

In linear mixed model analyses, adjusted for time and including a multiplicative interaction term of time with the donor body composition measure, no significant associations were found between any donor body composition measure and the annual change in recipient post-transplantation eGFR (donor BMI: *B* = 0.02, 95%CI -0.10; 0.13, *p* = 0.79; donor waist circumference: *B* = 0.01, 95%CI -0.03; 0.05, *p* = 0.54; donor skeletal muscle index: *B* = -0.002, 95%CI -0.05; 0.05, *p* = 0.94; donor skeletal muscle radiation attenuation: *B* = -0.01, 95%CI -0.06; 0.04, *p* = 0.65; donor visceral adipose tissue index: *B* = 0.002, 95%CI -0.01; 0.02, *p* = 0.76; donor subcutaneous adipose tissue index: *B* = -0.001, 95%CI -0.02; 0.01, *p* = 0.87; donor intramuscular adipose tissue index: *B* = -0.08, 95%CI -0.25; 0.09, *p* = 0.36; donor total abdominal adipose tissue index: *B* = 0.00, 95%CI -0.01; 0.01, *p* = 0.93) (**Table 2**). Annual change in recipient post-transplantation eGFR was lower in those with a kidney from donors who were overweight and simultaneously had a higher amount of intramuscular adipose tissue compared to those with a kidney from donors who were overweight with a lower amount of intramuscular adipose tissue (*B* = -1.19, 95%CI -2.29; -0.09, *p* = 0.04).

**Table 2:** Time-adjusted linear mixed model analyses of donor body composition measurements and post-transplantation kidney function trajectory.

	Recipient post- transplantation eGFR		
Donor body composition measurements	B	95% CI	p
Donor BMI	0.02	-0.10; 0.13	0.79
Donor waist circumference	0.01	-0.03; 0.05	0.54
Skeletal muscle index	-0.002	-0.05; 0.05	0.94
Skeletal muscle radiation attenuation	-0.01	-0.06; 0.04	0.65
Visceral adipose tissue index	0.002	-0.01; 0.02	0.76
Subcutaneous adipose tissue index	-0.001	-0.02; 0.01	0.87
Intramuscular adipose tissue index	-0.08	-0.25; 0.09	0.36
Total abdominal adipose tissue index	0.00	-0.01; 0.01	0.93

B: annual change in recipient post-transplantation eGFR for every 1-unit increase in body composition measurement.

Additional adjustments for donor female sex, donor age, donor mGFR, recipient female sex, recipient age, as well as multiplicative interaction terms of these independent variables with time, did not uncover a statistically significant association between donor body composition measurements and annual change in recipient post-transplantation eGFR (**Table 3**). Neither anthropometric measures of body composition (donor BMI: *B* = -0.01, 95%CI -0.13; 0.11, *p* = 0.88; donor waist circumference: *B* = 0.02, 95%CI -0.02; 0.06, *p* = 0.38), nor radiologic measures of body composition were associated with recipient post-transplantation eGFR (donor skeletal muscle index: *B* = -0.02, 95%CI -0.07; 0.04, *p* = 0.63; donor skeletal muscle radiation attenuation: *B* = -0.002, 95%CI -0.06; 0.06, *p* = 0.96; donor visceral adipose tissue index: *B* = -0.001, 95%CI -0.02; 0.02, *p* = 0.93; donor subcutaneous adipose tissue index: *B* = -0.001, 95%CI -0.02; 0.02, *p* = 0.94; donor intramuscular adipose tissue index: *B* = -0.12, 95%CI -0.29; 0.06, *p* = 0.19; donor total abdominal adipose tissue index: *B* = -0.001, 95%CI -0.01; 0.01, *p* = 0.89).

**Table 3:** Adjusted linear mixed model analyses of donor body composition measurements and post-transplantation kidney function trajectory.

	Recipient post- transplantation eGFR		
Donor body composition measurements	*B*	*95% CI*	*p*
Donor BMI, kg/m^2^	-0.01	-0.13; 0.11	0.88
Donor waist circumference, cm	0.02	-0.02; 0.06	0.38
Skeletal muscle index	-0.02	-0.07; 0.04	0.63
Skeletal muscle radiation attenuation	-0.002	-0.06; 0.06	0.96
Visceral adipose tissue index	-0.001	-0.02; 0.02	0.93
Subcutaneous adipose tissue index	-0.001	-0.02; 0.02	0.94
Intramuscular adipose tissue index	-0.12	-0.29; 0.06	0.19
Total abdominal adipose tissue index	-0.001	-0.01; 0.01	0.89

linear mixed models consist of: donor body composition measurement + time (years) + donor female sex + donor age (years) + donor measured glomerular filtration rate (ml/min) + recipient female sex + recipient age (years) + time*body composition measurement + time*donor female sex + time*donor age + time*donor measured glomerular filtration rate + time*recipient female sex + time*recipient age.

B: annual change in recipient post-transplantation eGFR for every 1-unit increase in body composition measurement.

Higher donor age was consistently significantly associated with lower recipient eGFR, although its interaction with time was not significantly associated with recipient eGFR (**Supplementary Tables S1**, **S2**).

Multiple post-transplant eGFR assessments were available for 161 (55%) recipients. Sensitivity analyses with only this subgroup of the study population did not substantially change the results, which are shown in **Supplementary Tables 4**, **5**.

## Discussion

We initially hypothesized that higher donor BMI, waist circumference, and radiologic fat tissue measurements are associated with a greater negative change/GFR reduction over time in recipients eGFR after transplantation. However, the results of this study show that, in donors with relatively “normal” body sizes, living kidney donor body composition is not associated with post-transplantation kidney function over time in kidney transplant recipients. Additionally, our findings revealed that annual change in recipient post-transplantation eGFR was lower in those with a kidney from donors who are overweight and simultaneously have a higher amount of intramuscular adipose tissue compared to overweight donors with a lower amount of intramuscular adipose tissue. While donor body composition appears to impact long-term kidney function in the donor, it does not exert a discernible effect on kidney function outcomes for the recipient in this specific study population.

To our knowledge, this is the first study to explore the relationship between living kidney donor body composition and recipient kidney function after living donor kidney transplantation. While this area is relatively unexplored in kidney transplantation, results from other fields of organ transplantation, such as liver transplantation, offer valuable perspectives. For instance, in liver transplantation, high muscle mass and quality in male donors were found to be a protective factor of allograft loss after living donor liver transplantation, while in female donors these factors did not affect allograft loss (23). Similarly, high intramuscular adipose tissue content among living liver donors was identified as an independent risk factor for 6-month graft survival (24). These associations are attributed to the secretion of myokines which influence adipose tissue mass and fat deposition in the liver (25, 26). It is possible that similar mechanisms may influence outcomes in kidney transplantation. Our study suggests that muscle quality in overweight living kidney donors affects recipient kidney function. Further research is needed to explore these potential relationships in kidney transplantation to better understand the underlying biological mechanisms.

In the context of living kidney donation, donor characteristics such as obesity and age have been shown to influence transplant outcomes. A recent systematic review and meta-analysis found that living kidney donor obesity (BMI > 30) affects the incidence of delayed graft function, but not the incidence of acute rejection in recipients (9). Moreover, obese donor recipient pairs were found to be at a higher risk for death censored graft loss, all-cause graft loss, early graft loss and delayed graft function compared to non-obese pairs (27). Other factors such as age also play an evident role in recipient outcomes. Age is not only closely related to body composition but can also independently impact kidney function (28–30). Our study shows that donor age seems to be a determinant of recipient post-transplant kidney function levels, although it was not associated with post-transplantation change in kidney function over time. A meta-analysis showed that one-year serum creatinine was significantly lower in kidney transplant recipients from donors aged <60 years compared to donors aged >60 years (9). Further exploration of these factors may enhance risk stratification strategies and optimize transplant outcomes, ultimately benefiting both donors and recipients alike.

The clinical implications of our results are multifaceted. Our findings suggest that, in the context of kidney transplantation with a graft from a living donor with a relatively “normal” body size, assessing living donor body composition may not be a critical determinant of post-transplant recipient kidney function. Instead, it may be more important to focus on other donor factors, such as age, to ensure post-transplant success. Obesity has been associated with peri- and postoperative complications such as wound infections, increased surgical blood loss and longer operation time (27). After donation, literature suggests that kidney donor body composition is associated with for example donor (long-term) kidney function, risk of developing end-stage kidney disease, and mortality (28, 31–34). Therefore, assessing and possibly improving donor body composition could still be of interest for the (long-term) health of the kidney donor, although further investigation is necessary to establish this link.

The study’s strength lies in its use of both conventional (anthropometric) techniques and radiologic measurements for body composition assessment. However, limitations include the retrospective design of the single center study in The Netherlands, the inability to adjust for race due to population homogeneity, and the exclusion of donors with a pre-donation BMI of ≥35 kg/m^2^, cautioning against generalizing the results to more diverse populations. Additionally, the absence of standardized normal or cut-off values for body composition indices hampers the comparability of these results with other studies. Therefore, establishing reference values for body composition indices should be a key focus of future research, both for donors and recipients. Nevertheless, the evidence provided supports the notion that transplanting kidneys from living donors with a BMI below 35 kg/m^2^ does not appear to affect recipient post-transplant kidney function, encouraging further exploration in larger, more diverse cohorts. In addition, the study not only expands knowledge within the specific context of kidney transplantation but also contributes to the broader field of body composition research by advocating for more comprehensive and sophisticated measurement techniques. This emphasis on methodological improvement holds the potential to enhance the accuracy and applicability of body composition assessments in diverse clinical and research settings and can contribute to better outcomes for both living kidney donors and recipients.

In conclusion, this study, using a combination of anthropometric and radiologic measurements, provides insights into the association between living kidney donor body composition and kidney function in kidney transplant recipients. These results stem from a cohort of living kidney donors with relatively “normal” body sizes, suggesting that in the context of transplanting kidneys from living donors with a BMI below 35 kg/m^2^, pre-donation body composition does not affect post-transplantation recipient kidney function. These results invite further investigation in larger, more heterogenous study populations to refine our understanding of these relationships.

## Data Availability

The data analyzed in this study is subject to the following licenses/restrictions: TransplantLines datasets may be retrieved upon reasonable request. Requests to access these datasets should be directed to r.pol@umcg.nl.

## References

[1] Purnell TS Auguste P Crews DC Lamprea-Montealegre J Olufade T Greer R. Comparison of life participation activities among adults treated by hemodialysis, peritoneal dialysis, and kidney transplantation: a systematic review. Am J Kidney Dis. (2013) 62:953–73. doi: 10.1053/j.ajkd.2013.03.022 PMC3809150 23725972

[2] Cozzi E Biancone L López-Fraga M Nanni-Costa A. Long-term outcome of living kidney donation: position paper of the european committee on organ transplantation, council of Europe. Transplantation. (2016) 100:270–1. doi: 10.1097/TP.0000000000000994 26528770

[3] Hart A Smith JM Skeans MA Gustafson SK Wilk AR Robinson A. OPTN/SRTR 2016 annual data report: kidney. Am J Transplant. (2018) 18 Suppl 1:18–113. doi: 10.1111/ajt.14557 29292608 PMC5772947

[4] Mange KC Joffe MM Feldman HI. Effect of the use or nonuse of long-term dialysis on the subsequent survival of renal transplants from living donors. N Engl J Med. (2001) 344:726–31. doi: 10.1056/NEJM200103083441004 11236776

[5] Reese PP Boudville N Garg AX. Living kidney donation: outcomes, ethics, and uncertainty. Lancet. (2015) 385:2003–13. doi: 10.1016/S0140-6736(14)62484-3 26090646

[6] Bentata Y. Obesity in living-donor kidney transplant: what risks for the donor and the recipient? Exp Clin Transplant. (2021) 19:287–96. doi: 10.6002/ect.2020.0074 32635886

[7] García-Carro C Vergara A Bermejo S Azancot MA Sellarés J Soler MJ. A nephrologist perspective on obesity: from kidney injury to clinical management. Front Med (Lausanne). (2021) 8:655871. doi: 10.3389/fmed.2021.655871 33928108 PMC8076523

[8] Naik AS Zhong Y Parasuraman R Doshi M Norman S Lu Y. The temporal and long-term impact of donor body mass index on recipient outcomes after kidney transplantation - a retrospective study. Transpl Int. (2020) 33:59–67. doi: 10.1111/tri.13505 31478267

[9] Bellini MI Nozdrin M Pengel L Knight S Papalois V. How good is a living donor? Systematic review and meta-analysis of the effect of donor demographics on post kidney transplant outcomes. J Nephrol. (2022) 35:807–20. doi: 10.1007/s40620-021-01231-7 PMC8995249 35072936

[10] Kanbay M Copur S Ucku D Zoccali C. Donor obesity and weight gain after transplantation: two still overlooked threats to long-term graft survival. Clin Kidney J. (2023) 16:254–61. doi: 10.1093/ckj/sfac216 PMC9900567 36755848

[11] Mandelbrot D Pavlakis M Danovitch G Johnson SR Karp SJ Khwaja K. The medical evaluation of living kidney donors: A survey of US transplant centers. Am J Transplant. (2007) 7:2333–43. doi: 10.1111/j.1600-6143.2007.01932.x 17845567

[12] Lee DH Keum N Hu FB Orav EJ Rimm EB Willett WC. Predicted lean body mass, fat mass, and all cause and cause specific mortality in men: prospective US cohort study. BMJ. (2018) 362:k2575. doi: 10.1136/bmj.k2575 29970408 PMC6028901

[13] Karakizlis H Trudel N Brose A Reinisch A Reichert M Hecker A. Sarcopenia of kidney transplant recipients as a predictive marker for reduced graft function and graft survival after kidney transplantation. Langenbecks Arch Surg. (2023) 408:103. doi: 10.1007/s00423-023-02836-1 36826595 PMC9958183

[14] Druckmann I Yashar H Schwartz D Schwartz IF Goykhman Y Kliuk Ben-Bassat O. Presence of sarcopenia before kidney transplantation is associated with poor outcomes. Am J Nephrol. (2022) 53:427–34. doi: 10.1159/000524774 PMC9393828 35584614

[15] Morel A Ouamri Y Canouï-Poitrine F Mulé S Champy CM Ingels A. Myosteatosis as an independent risk factor for mortality after kidney allograft transplantation: a retrospective cohort study. J Cachexia Sarcopenia Muscle. (2022) 13:386–96. doi: 10.1002/jcsm.12853 PMC8818595 34738343

[16] Zhu Q Scherer PE. Immunologic and endocrine functions of adipose tissue: implications for kidney disease. Nat Rev Nephrol. (2018) 14:105–20. doi: 10.1038/nrneph.2017.157 29199276

[17] Helal I Fick-Brosnahan G Reed-Gitomer B Schrier R. Glomerular hyperfiltration: definitions, mechanisms and clinical implications. Nat Rev Nephrol. (2012) 8:293–300. doi: 10.1038/nrneph.2012.19 22349487

[18] Chagnac A Zingerman B Rozen-Zvi B Herman-Edelstein M. Consequences of glomerular hyperfiltration: the role of physical forces in the pathogenesis of chronic kidney disease in diabetes and obesity. Nephron. (2019) 143:38–42. doi: 10.1159/000499486 30947190

[19] Eisenga MF Gomes-Neto AW van Londen M Ziengs AL Douwes RM Stam SP. Rationale and design of TransplantLines: a prospective cohort study and biobank of solid organ transplant recipients. BMJ Open. (2018) 8:e024502. doi: 10.1136/bmjopen-2018-024502 PMC6318532 30598488

[20] Mourtzakis M Prado CM Lieffers JR Reiman T McCargar LJ Baracos VE. A practical and precise approach to quantification of body composition in cancer patients using computed tomography images acquired during routine care. Appl Physiol Nutr Metab. (2008) 33:997–1006. doi: 10.1139/H08-075 18923576

[21] Westenberg LB Zorgdrager M Swaab TDA van Londen M Bakker SJL Leuvenink HGD. Reference values for low muscle mass and myosteatosis using tomographic muscle measurements in living kidney donors. Sci Rep. (2023) 13:5835. doi: 10.1038/s41598-023-33041-1 37037940 PMC10086018

[22] Inker LA Eneanya ND Coresh J Tighiouart H Wang D Sang Y. Chronic Kidney Disease Epidemiology Collaboration. New creatinine- and cystatin C–based equations to estimate GFR without race. N Engl J Med. (2021) 385:1737–49. doi: 10.1056/NEJMoa2102953 PMC8822996 34554658

[23] Miyachi Y Kaido T Hirata M Iwamura S Yao S Shirai H. The combination of a male donor's high muscle mass and quality is an independent protective factor for graft loss after living donor liver transplantation. Am J Transplant. (2020) 20:3401–12. doi: 10.1111/ajt.15884 32243072

[24] Tomiyama T Harada N Toshima T Nakayama Y Toshida K Morinaga A. Donor skeletal muscle quality affects graft mortality after living donor liver transplantation- A single center, retrospective study. Transpl Int. (2022) 35:10723. doi: 10.3389/ti.2022.10723 36568139 PMC9784912

[25] Merli M Lattanzi B Aprile F. Sarcopenic obesity in fatty liver. Curr Opin Clin Nutr Metab Care. (2019) 22:185–90. doi: 10.1097/MCO.0000000000000558 30893090

[26] Kim G Kim JH. Impact of skeletal muscle mass on metabolic health. Endocrinol Metab (Seoul). (2020) 35:1–6. doi: 10.3803/EnM.2020.35.1.1 32207258 PMC7090295

[27] Tjeertes E Hoeks S Beks S Valentijn T Hoofwijk A Stolker R. Obesity—A risk factor for postoperative complications in general surgery. BMC Anesthesiol. (2015) 15:112. doi: 10.1186/s12871-015-0096-7 26228844 PMC4520073

[28] Jarrar F Tennankore KK Vinson AJ. Combined donor-recipient obesity and the risk of graft loss after kidney transplantation. Transpl Int. (2022) 35:10656. doi: 10.3389/ti.2022.10656 36247488 PMC9556700

[29] Westenberg LB Pol RA van der Weijden J de Borst MH Bakker SJL van Londen M. Central body fat distribution and kidney function after living kidney donation. Clin J Am Soc Nephrol. (2024) 19(4):503–13. doi: 10.2215/CJN.0000000000000403 PMC11020429 38190119

[30] Suetta C Haddock B Alcazar J Noerst T Hansen OM Ludvig H. The Copenhagen Sarcopenia Study: lean mass, strength, power, and physical function in a Danish cohort aged 20-93 years. J Cachexia Sarcopenia Muscle. (2019) 10:1316–29. doi: 10.1002/jcsm.12477 PMC6903448 31419087

[31] Grams ME Sang Y Levey AS Matsushita K Ballew S Chang AR. Kidney-failure risk projection for the living kidney-donor candidate. N Engl J Med. (2016) 374:411–21. doi: 10.1056/NEJMoa1510491 PMC4758367 26544982

[32] Locke JE Reed RD Massie A MacLennan PA Sawinski D Kumar V. Obesity increases the risk of end-stage renal disease among living kidney donors. Kidney Int. (2017) 91:699–703. doi: 10.1016/j.kint.2016.10.014 28041626 PMC5313335

[33] Locke JE Reed RD Massie A MacLennan PA Sawinski D Kumar V. Obesity and long-term mortality risk among living kidney donors. Surgery. (2019) 166:205–8. doi: 10.1016/j.surg.2019.03.016 PMC6660998 31072668

[34] Petermann-Rocha F Balntzi V Gray SR Lara J Ho FK Pell JP. Global prevalence of sarcopenia and severe sarcopenia: a systematic review and meta-analysis. J Cachexia Sarcopenia Muscle. (2022) 13:86–99. doi: 10.1002/jcsm.12783 34816624 PMC8818604

