# Supplementary material for: Analyzing body composition in living kidney donors: impact on post-transplant kidney function
Source: Front Nephrol. 2024 Nov 25;4:1467669. doi: 10.3389/fneph.2024.1467669 (PMC11625803; doi:10.3389/fneph.2024.1467669)
Supplement: Supplementary file 1 [file DataSheet1.docx]

**Supplemental Digital Content**

Supplemental Figure S1. Visual overvies of the number of consecutive post-transplant eGFR measurements per recipient.


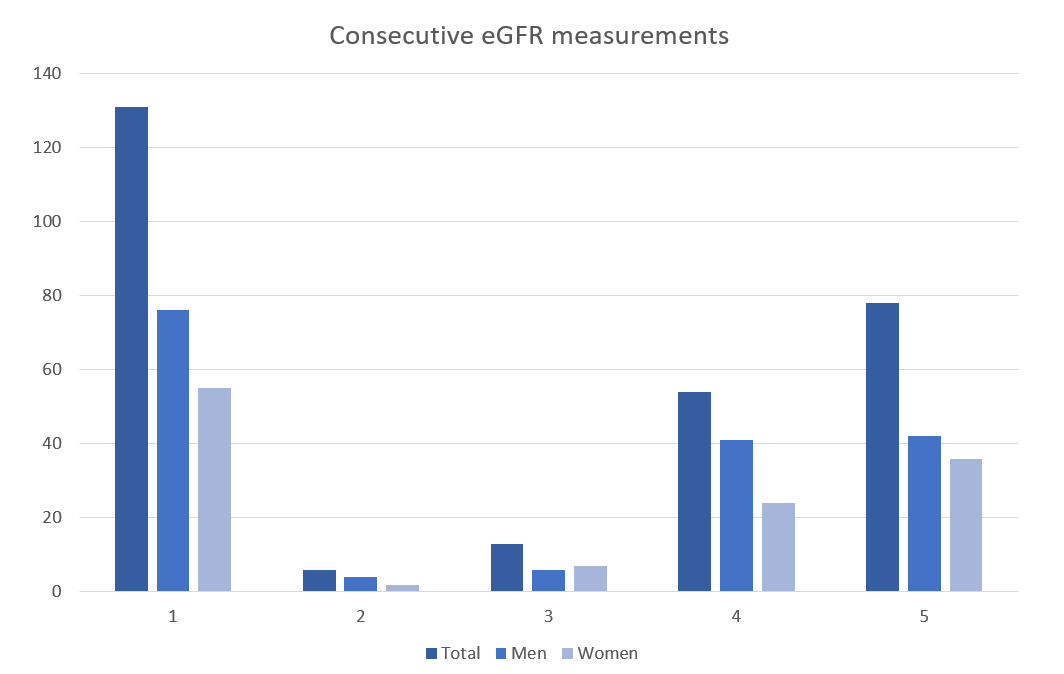


Supplemental Table 1. Linear mixed model analyses of anthropometric donor body composition measurements and post-transplantation kidney function trajectory.

|  | **Recipient post-transplantation eGFR** | | | | | |
| --- | --- | --- | --- | --- | --- | --- |
|  | Time-adjusted model | | | Adjusted model | | |
| **Fixed effects** | *B* | *95% CI* | *p* | *B* | *95% CI* | *p* |
| Donor BMI | -0.32 | -1.04; 0.40 | 0.39 | -0.33 | -1.00; 0.35 | 0.34 |
| Time | -0.19 | -3.19; 2.80 | 0.90 | -2.98 | -7.26; 1.29 | 0.17 |
| Donor female sex |  |  |  | 1.32 | -3.22; 5.86 | 0.57 |
| Donor age |  |  |  | -0.49 | -0.69; -0.28 | <0.001 |
| Donor mGFR |  |  |  | 0.16 | 0.03; 0.28 | 0.01 |
| Recipient female sex |  |  |  | -1.76 | -5.77; 2.25 | 0.39 |
| Recipient age |  |  |  | -0.09 | -0.25; 0.06 | 0.25 |
| Time * donor BMI | 0.02 | -0.10; 0.13 | 0.79 | -0.01 | -0.13; 0.11 | 0.88 |
| Time * donor female sex |  |  |  | 0.22 | -0.58; 1.02 | 0.60 |
| Time * donor age |  |  |  | -0.01 | -0.05; 0.03 | 0.55 |
| Time * donor mGFR |  |  |  | 0.01 | -0.01; 0.03 | 0.28 |
| Time * recipient female sex |  |  |  | -0.49 | -1.20; 0.22 | 0.18 |
| Time * recipient age |  |  |  | 0.06 | 0.03; 0.08 | <0.001 |
| Donor waist circumference | -0.11 | -0.36; 0.14 | 0.38 | -0.11 | -0.36; 0.14 | 0.40 |
| Time | -0.94 | -4.33; 2.46 | 0.59 | -2.40 | -7.18; 2.37 | 0.32 |
| Donor female sex |  |  |  | 0.88 | -4.51; 6.26 | 0.75 |
| Donor age |  |  |  | -0.48 | -0.72; -0.24 | <0.001 |
| Donor mGFR |  |  |  | 0.18 | 0.04; 0.32 | 0.02 |
| Recipient female sex |  |  |  | -1.59 | -6.15; 2.97 | 0.49 |
| Recipient age |  |  |  | -0.12 | -0.30; 0.07 | 0.22 |
| Time * donor waist circumference | 0.01 | -0.03; 0.05 | 0.54 | 0.02 | -0.02; 0.06 | 0.38 |
| Time * donor female sex |  |  |  | 0.35 | -0.52; 1.21 | 0.43 |
| Time * donor age |  |  |  | -0.03 | -0.07; 0.02 | 0.24 |
| Time * donor mGFR |  |  |  | -0.004 | -0.03; 0.02 | 0.77 |
| Time * recipient female sex |  |  |  | -0.56 | -1.32; 0.20 | 0.15 |
| Time * recipient age |  |  |  | 0.05 | 0.02; 0.08 | <0.001 |

Random effects: intercept

Supplemental Table 2. Linear mixed model analyses of radiologic donor body composition measurements and post-transplantation kidney function trajectory.

|  | **Recipient post-transplantation eGFR** | | | | | |
| --- | --- | --- | --- | --- | --- | --- |
|  | Time-adjusted model | | | Adjusted model | | |
| **Fixed effects** | *B* | *95% CI* | *p* | *B* | *95% CI* | *p* |
| Donor SMI | 0.06 | -0.24; 0.35 | 0.70 | -0.18 | -0.53; 0.17 | 0.32 |
| Time | 0.28 | -2.03; 2.59 | 0.81 | -2.52 | -7.03; 1.98 | 0.27 |
| Donor female sex |  |  |  | -0.30 | -5.69; 5.08 | 0.91 |
| Donor age |  |  |  | -0.50 | -0.71; -0.30 | <0.001 |
| Donor mGFR |  |  |  | 0.15 | 0.03; 0.27 | 0.01 |
| Recipient female sex |  |  |  | -1.94 | -5.93; 2.05 | 0.34 |
| Recipient age |  |  |  | -0.09 | -0.24; 0.07 | 0.27 |
| Time * donor SMI | -0.002 | -0.05; 0.05 | 0.94 | -0.02 | -0.07; 0.04 | 0.63 |
| Time * donor female sex |  |  |  | 0.05 | -0.89; 1.00 | 0.91 |
| Time * donor age |  |  |  | -0.01 | -0.05; 0.03 | 0.56 |
| Time * donor mGFR |  |  |  | 0.01 | -0.01; 0.03 | 0.24 |
| Time * recipient female sex |  |  |  | -0.48 | -1.19; 0.23 | 0.19 |
| Time * recipient age |  |  |  | 0.06 | 0.03; 0.08 | <0.001 |
| Donor SMRA | 0.56 | 0.24; 0.88 | <0.001 | 0.15 | -0.19; 0.48 | 0.39 |
| Time | 0.86 | -1.81; 3.54 | 0.53 | -3.02 | -8.85; 2.82 | 0.31 |
| Donor female sex |  |  |  | 1.63 | -2.95; 6.21 | 0.48 |
| Donor age |  |  |  | -0.45 | -0.68; -0.23 | <0.001 |
| Donor mGFR |  |  |  | 0.15 | 0.03; 0.27 | 0.02 |
| Recipient female sex |  |  |  | -1.98 | -6.00; 2.04 | 0.33 |
| Recipient age |  |  |  | -0.10 | -0.26; 0.05 | 0.20 |
| Time * donor SMRA | -0.01 | -0.06; 0.04 | 0.65 | -0.002 | -0.06; 0.06 | 0.96 |
| Time * donor female sex |  |  |  | 0.20 | -0.61; 1.02 | 0.63 |
| Time * donor age |  |  |  | -0.02 | -0.06; 0.03 | 0.49 |
| Time * donor mGFR |  |  |  | 0.01 | -0.01; 0.03 | 0.30 |
| Time * recipient female sex |  |  |  | -0.48 | -1.19; 0.24 | 0.19 |
| Time * recipient age |  |  |  | 0.06 | 0.03; 0.09 | <0.001 |
| Donor VATi | -0.12 | -0.21; -0.03 | 0.01 | -0.03 | -0.13; 0.07 | 0.54 |
| Time | 0.11 | -0.61; 0.82 | 0.77 | -3.07 | -6.95; 0.81 | 0.12 |
| Donor female sex |  |  |  | 0.76 | -4.07; 5.59 | 0.76 |
| Donor age |  |  |  | -0.48 | -0.70; -0.26 | <0.001 |
| Donor mGFR |  |  |  | 0.14 | 0.02; 0.26 | 0.02 |
| Recipient female sex |  |  |  | -1.95 | -5.96; 2.05 | 0.34 |
| Recipient age |  |  |  | -0.09 | -0.25; 0.06 | 0.25 |
| Time * donor VATi | 0.002 | -0.01; 0.02 | 0.76 | -0.001 | -0.02; 0.02 | 0.93 |
| Time * donor female sex |  |  |  | 0.21 | -0.62; 1.04 | 0.62 |
| Time * donor age |  |  |  | -0.01 | -0.06; 0.03 | 0.55 |
| Time * donor mGFR |  |  |  | 0.01 | -0.01; 0.03 | 0.29 |
| Time * recipient female sex |  |  |  | -0.50 | -1.22; 0.22 | 0.17 |
| Time * recipient age |  |  |  | 0.06 | 0.03; 0.08 | <0.001 |
| Donor SATi | -0.04 | -0.13; 0.05 | 0.36 | -0.04 | -0.12; 0.05 | 0.39 |
| Time | 0.26 | -0.58; 1.10 | 0.54 | -3.17 | -7.04; 0.70 | 0.11 |
| Donor female sex |  |  |  | 2.28 | -2.79; 7.35 | 0.38 |
| Donor age |  |  |  | -0.50 | -0.70; -0.29 | <0.001 |
| Donor mGFR |  |  |  | 0.14 | 0.03; 0.26 | 0.02 |
| Recipient female sex |  |  |  | -1.80 | -5.81; 2.21 | 0.38 |
| Recipient age |  |  |  | -0.09 | -0.25; 0.06 | 0.24 |
| Time * donor SATi | -0.001 | -0.02; 0.01 | 0.87 | -0.001 | -0.02; 0.02 | 0.94 |
| Time * donor female sex |  |  |  | 0.24 | -0.68; 1.17 | 0.61 |
| Time * donor age |  |  |  | -0.01 | -0.05; 0.03 | 0.53 |
| Time * donor mGFR |  |  |  | 0.01 | -0.01; 0.03 | 0.28 |
| Time * recipient female sex |  |  |  | -0.49 | -1.21; 0.22 | 0.17 |
| Time * recipient age |  |  |  | 0.06 | 0.03; 0.08 | <0.001 |
| Donor IMATi | -0.84 | -1.83; 0.16 | 0.10 | 0.33 | -0.64; 1.31 | 0.50 |
| Time | 0.54 | -0.27; 1.34 | 0.19 | -3.30 | -7.18; 0.57 | 0.09 |
| Donor female sex |  |  |  | 1.36 | -3.20; 5.91 | 0.56 |
| Donor age |  |  |  | -0.53 | -0.75; -0.31 | <0.001 |
| Donor mGFR |  |  |  | 0.14 | 0.02; 0.25 | 0.02 |
| Recipient female sex |  |  |  | -2.02 | -6.04; 1.99 | 0.32 |
| Recipient age |  |  |  | -0.09 | -0.25; 0.06 | 0.25 |
| Time * donor IMATi | -0.08 | -0.25; 0.09 | 0.36 | -0.12 | -0.29; 0.06 | 0.19 |
| Time * donor female sex |  |  |  | 0.24 | -0.56; 1.04 | 0.55 |
| Time * donor age |  |  |  | -0.01 | -0.05; 0.04 | 0.83 |
| Time * donor mGFR |  |  |  | 0.01 | -0.01; 0.03 | 0.23 |
| Time * recipient female sex |  |  |  | -0.46 | -1.17; 0.25 | 0.20 |
| Time * recipient age |  |  |  | 0.06 | 0.03; 0.08 | <0.001 |
| Donor TATi | -0.06 | -0.12; -0.01 | 0.03 | -0.02 | -0.07; 0.03 | 0.42 |
| Time | 0.15 | -0.78; 1.08 | 0.75 | -3.11 | -6.98; 0.77 | 0.12 |
| Donor female sex |  |  |  | 1.44 | -3.12; 6.01 | 0.53 |
| Donor age |  |  |  | -0.48 | -0.70; -0.27 | <0.001 |
| Donor mGFR |  |  |  | 0.14 | 0.03; 0.26 | 0.02 |
| Recipient female sex |  |  |  | -1.87 | -5.88; 2.13 | 0.36 |
| Recipient age |  |  |  | -0.09 | -0.25; 0.06 | 0.24 |
| Time * donor TATi | 0.00 | -0.01; 0.01 | 0.93 | -0.001 | -0.01; 0.01 | 0.89 |
| Time * donor female sex |  |  |  | 0.23 | -0.58; 1.05 | 0.57 |
| Time * donor age |  |  |  | -0.01 | -0.05; 0.03 | 0.56 |
| Time * donor mGFR |  |  |  | 0.01 | -0.01; 0.03 | 0.28 |
| Time * recipient female sex |  |  |  | -0.51 | -1.22; 0.21 | 0.17 |
| Time * recipient age |  |  |  | 0.06 | 0.03; 0.08 | <0.001 |

Random effects: intercept

Linear mixed models consist of: donor body composition measurement + time (years) + donor female sex + donor age (years) + donor measured glomerular filtration rate (ml/min) + recipient female sex + recipient age (years) + time*body composition measurement + time*donor female sex + time*donor age + time*donor measured glomerular filtration rate + time*recipient female sex + time*recipient age.

B: annual change in recipient post-transplantation eGFR for every 1-unit increase in body composition measurement.

Supplemental Table 3. Linear mixed model analyses of donor body composition measurements and post-transplantation kidney function trajectory, per BMI category.

|  | **Recipient post-transplantation eGFR** | | | | | |
| --- | --- | --- | --- | --- | --- | --- |
|  | **Time-adjusted model** | | | **Adjusted model** | | |
| **Donor body composition measurements** | *B* | *95% CI* | *p* | *B* | *95% CI* | *p* |
| **Fixed effects** |  |  |  |  |  |  |
| Donor waist circumference  High BMI (≥25 kg/m^2^)  Low BMI (<25 kg/m^2^) | 0.06  -0.02 | -0.02; 0.13  -0.11; 0.07 | 0.14  0.66 | 0.02  0.04 | -0.06; 0.10  -0.05; 0.14 | 0.58  0.38 |
| Skeletal muscle index  High BMI (≥25 kg/m^2^)  Low BMI (<25 kg/m^2^) | 0.03  -0.04 | -0.03; 0.09  -0.13; 0.05 | 0.35  0.35 | -0.04  0.01 | -0.13; 0.04  -0.10; 0.12 | 0.33  0.82 |
| Skeletal muscle radiation attenuation  High BMI (≥25 kg/m^2^)  Low BMI (<25 kg/m^2^) | -0.01  -0.05 | -0.09; 0.07  -0.13; 0.04 | 0.85  0.31 | 0.02  -0.05 | -0.06; 0.11  -0.15; 0.05 | 0.58  0.29 |
| Visceral adipose tissue index  High BMI (≥25 kg/m^2^)  Low BMI (<25 kg/m^2^) | 0.01  0.01 | -0.02; 0.03  -0.03; 0.04 | 0.56  0.78 | -0.02  0.02 | -0.05; 0.01  -0.02; 0.06 | 0.17  0.30 |
| Subcutaneous adipose tissue index  High BMI (≥25 kg/m^2^)  Low BMI (<25 kg/m^2^) | -0.01  0.02 | -0.03; 0.01  -0.01; 0.04 | 0.37  0.29 | 0.02  0.01 | -0.01; 0.05  -0.03; 0.04 | 0.19  0.75 |
| Intramuscular adipose tissue index  High BMI (≥25 kg/m^2^)  Low BMI (<25 kg/m^2^) | -0.07  -0.08 | -0.29; 0.15  -0.48; 0.31 | 0.53  0.68 | -0.11  -0.14 | -0.33; 0.12  -0.58; 0.31 | 0.36  0.55 |
| Total abdominal adipose tissue index  High BMI (≥25 kg/m^2^)  Low BMI (<25 kg/m^2^) | -0.002  0.01 | -0.02; 0.02  -0.01; 0.03 | 0.82  0.28 | -0.001  0.01 | -0.02; 0.02  -0.01; 0.03 | 0.93  0.44 |

Random effects: intercept

Linear mixed models consist of: donor body composition measurement + time (years) + donor female sex + donor age (years) + donor measured glomerular filtration rate (ml/min) + recipient female sex + recipient age (years) + time*body composition measurement + time*donor female sex + time*donor age + time*donor measured glomerular filtration rate + time*recipient female sex + time*recipient age.

B: annual change in recipient post-transplantation eGFR for every 1-unit increase in body composition measurement.

Supplemental Table 4. Time-adjusted linear mixed model analyses of donor body composition measurements and post-transplantation kidney function trajectory in a subgroup of recipients with multiple eGFR assessments.

|  | **Recipient post-transplantation eGFR** | | |
| --- | --- | --- | --- |
| **Donor body composition measurements** | B | 95% CI | p |
| Donor BMI | 0.03 | -0.10; 0.17 | 0.63 |
| Donor waist circumference | 0.01 | -0.03; 0.06 | 0.55 |
| Skeletal muscle index | 0.03 | -0.03; 0.09 | 0.34 |
| Skeletal muscle radiation attenuation | -0.003 | -0.06; 0.06 | 0.92 |
| Visceral adipose tissue index | 0.002 | -0.02; 0.02 | 0.85 |
| Subcutaneous adipose tissue index | -0.01 | -0.02; 0.01 | 0.40 |
| Intramuscular adipose tissue index | -0.16 | -0.34; 0.03 | 0.10 |
| Total abdominal adipose tissue index | -0.002 | -0.01; 0.01 | 0.63 |

B: annual change in recipient post-transplantation eGFR for every 1-unit increase in body composition measurement.

Supplemental Table 5. Adjusted linear mixed model analyses of donor body composition measurements and post-transplantation kidney function trajectory in a subgroup of recipients with multiple eGFR assessments.

|  | **Recipient post-transplantation eGFR** | | |
| --- | --- | --- | --- |
| **Donor body composition measurements** | *B* | *95% CI* | *p* |
| Donor BMI, kg/m^2^ | 0.03 | -0.11; 0.17 | 0.68 |
| Donor waist circumference, cm | 0.03 | -0.02; 0.07 | 0.31 |
| Skeletal muscle index | 0.01 | -0.07; 0.08 | 0.84 |
| Skeletal muscle radiation attenuation | -0.002 | -0.07; 0.07 | 0.96 |
| Visceral adipose tissue index | -0.004 | -0.03; 0.02 | 0.68 |
| Subcutaneous adipose tissue index | -0.002 | -0.02; 0.02 | 0.83 |
| Intramuscular adipose tissue index | -0.20 | -0.39; 0.00 | 0.05 |
| Total abdominal adipose tissue index | -0.003 | -0.01; 0.01 | 0.65 |

linear mixed models consist of: donor body composition measurement + time (years) + donor female sex + donor age (years) + donor measured glomerular filtration rate (ml/min) + recipient female sex + recipient age (years) + time*body composition measurement + time*donor female sex + time*donor age + time*donor measured glomerular filtration rate + time*recipient female sex + time*recipient age.

B: annual change in recipient post-transplantation eGFR for every 1-unit increase in body composition measurement.

Supplemental Table 6. Overview of donors across different BMI classifications.

| **BMI (kg/m^2^)** | **WHO classification** | **No. of donors** |
| --- | --- | --- |
| < 18.5 | Underweight | 2 |
| 18.5 – 24.9 | Normal range | 70 |
| 25.0 – 29.9 | Overweight | 78 |
| 30.0 – 34.9 | Obesity | 11 |
| ≥ 35 | Severe obesity | 0 |

Supplemental Table 7. Linear mixed model analyses with BMI and intramuscular adipose tissue index.

|  | **Recipient post-transplantation eGFR** | | |
| --- | --- | --- | --- |
| **Donor body composition categories** | B | 95% CI | p |
| Normal BMI and intramuscular adipose tissue index > mean (13.85) | 1.20 | -0.59; 2.98 | 0.19 |
| BMI ≥25 kg/m^2^ and intramuscular adipose tissue index > mean (13.85) | -1.19 | -2.29; -0.09 | 0.04 |
